# Phosphorescent Imaging of Living Cells Using a Cyclometalated Iridium(III) Complex

**DOI:** 10.1371/journal.pone.0055751

**Published:** 2013-02-14

**Authors:** Dik-Lung Ma, Hai-Jing Zhong, Wai-Chung Fu, Daniel Shiu-Hin Chan, Hiu-Yee Kwan, Wang-Fun Fong, Lai-Hon Chung, Chun-Yuen Wong, Chung-Hang Leung

**Affiliations:** 1 Department of Chemistry, Hong Kong Baptist University, Kowloon Tong, Hong Kong, China; 2 State Key Laboratory of Quality Research in Chinese Medicine, Institute of Chinese Medical Sciences, University of Macau, Macao, China; 3 Center for Cancer and Inflammation Research, School of Chinese Medicine, Hong Kong Baptist University, Kowloon, Hong Kong, China; 4 Department of Biology and Chemistry, City University of Hong Kong, Tat Chee Avenue, Kowloon, Hong Kong SAR, People's Republic of China; NIH, United States of America

## Abstract

A cell permeable cyclometalated iridium(III) complex has been developed as a phosphorescent probe for cell imaging. The iridium(III) solvato complex [Ir(phq)_2_(H_2_O]_2_)] preferentially stains the cytoplasm of both live and dead cells with a bright luminescence.

## Introduction

Luminescence imaging of biological specimens using non-invasive probes is a basic technique in life and biomedical sciences for studying the morphologic characteristics of tissue at high resolution [1]–[5]. Since the cell is the primary structural and functional unit of all known living organisms, the morphological aberration of certain cell types can lead to various diseases such as sickle cell anemia [6], [7]. Consequently, a great deal of attention has been invested into the development of luminescent probes for live cell imaging in recent years. Currently, organic dyes constitute the majority of the most commonly-used fluorescent probes [8]. However, organic dyes can be subject to various drawbacks, including small Stokes shift values and short luminescence lifetimes [9]–[11]. In this context, luminescent transition metal complexes have arisen as viable alternatives to organic fluorophores for sensing and imaging applications due to the following advantages: [12]–[36] (i) tunable excitation and emission maxima over the visible region without the need for lengthy synthetic protocols; (ii) tunable emission energies by modification of the ancillary ligands; (iii) large Stokes shift for facile separation of excitation and emission wavelengths and elimination of self-quenching; (iv) relatively long phosphorescence lifetimes that can mitigate a short-lived autofluorescence background through the use of time-resolved spectroscopy which offers high selectivity; and (v) good solubility in aqueous solution (containing <0.01% organic solvent).

In eukaryotes, the cytoplasm is an aqueous fluid that primarily consists of a transparent substance termed hyaloplasm or cytosol. Numerous life processes take place within the cytoplasm, including protein synthesis, metabolic reactions, and cellular signaling. However, only a few phosphorescent metal complexes have been developed for cytoplasmic staining. For example, Coogan and co-workers have reported a series of Re(I) complexes of type fac-[Re(bisim)L(CO)_3_]^+^ containing highly lipophilic esters of 3-hydroxymethylpyridine as luminescence agents that selectively distribute in membranes and membrane structures within the cytoplasm of living cells [35]. Barton and co-workers investigated a series of phosphorescent ruthenium(II) complexes with different ancillary ligands that selectively stain the cytoplasm [37]. The groups of Li and Lo have developed a series of cationic iridium(III) complexes as phosphorescent probes for luminescence staining of the cytoplasm of living cells [29], [38]–[40].

Iridium(III) complexes with d^6^ electronic structures often possess excellent photophysical properties such as tunable excitation and emission wavelengths (from blue to red), high luminescent quantum yields, and relatively long phosphorescence lifetimes [41], [42]. Iridium complexes have received considerable attention in inorganic photochemistry [43]–[48], phosphorescent materials for optoelectronics [49]–[60], chemosensors [61]–[66], biolabeling[67]–[69], live cell imaging [29], [70]–[72], and in vivo tumor imaging [73]. As part of our continuous efforts, the cyclometalated iridium(III) solvato complex [Ir(ppy)_2_(solv)_2_]^+^ has been utilized as a selective luminescent switch-on probe for histidine/histidine-rich proteins and a dye for protein staining in sodium dodecyl sulfate polyacrylamide gels [74]. Subsequently, Li and co-workers reported iridium(III) solvato complex [Ir(ppy)_2_(DMSO)_2_]^+^ as a luminescence agent for imaging live cell nuclei [75]. Thus, we were interested to investigate the effect of varying the extent of conjugation of the C^∧^N co-ligand on the photophysical properties of this type of complex. We herein report the application of iridium(III) solvato complex [Ir(phq)_2_(solv)_2_]^+^ (1) for the detection of histidine/histidine-rich proteins and for luminescence imaging in cells. We demonstrate that the complex is successfully taken up by both living and dead cells and can function as a selective luminescent probe for cytoplasmic staining.

The luminescence response of complex 1 to various natural amino acids was investigated (Figure 3). Complex 1 is non-emissive in aqueous buffered solution in the absence of analyte. In the presence of histidine, complex 1 exhibits an intense phosphorescence emission at λ_max_ = 598 nm. No significant change in the emission of the complex 1 was observed upon the addition of other natural amino acids (Figure 3). This result indicates that complex 1 displays a high degree of selectivity for histidine over other amino acids. Furthermore, the emission maxima of 1 falls on the boundary of the near-infrared (NIR) “optical window” (600–900 nm), which is a region where the absorbance of photons by biological tissues decreases to a minimum [66]. This suggests that complex 1 may be potentially developed for in vivo imaging applications. By comparison, the previously reported iridium(III) complex [Ir(ppy)_2_(solv)_2_]^+^ (3) utilized for cellular staining emits green phosphorescence at a shorter wavelength of 505 nm in the presence of histidine, which is outside the optical window [74], [75].

We next studied the luminescence response of complex 1 with bovine serum albumin (BSA) and calf-thymus DNA (ct DNA). Complex 1 displayed an intense luminescence upon interaction with the histidine-rich BSA, but was only weakly emissive in the presence of ct DNA (Figure 4). Furthermore, the change in luminescence intensity of complex 1 upon the addition of various amounts (12.5−100 µM) of histidine or BSA was investigated. The results showed that BSA or histidine were able to induce significant luminescence enhancements in complex 1 (Figure 5). In combination with previously published reports [74], [76], we propose that the labile solvato co-ligands of complex 1 are displaced by the imidazole N-donor moieties of histidine residues via coordinative bond formation. This shelters the metal center within the hydrophobic environment of the protein, reducing solvent-mediated non-radiative decay of the excited state and thereby enhancing the phosphorescence of complex 1.

## Results and Discussion

**Figure 1:**
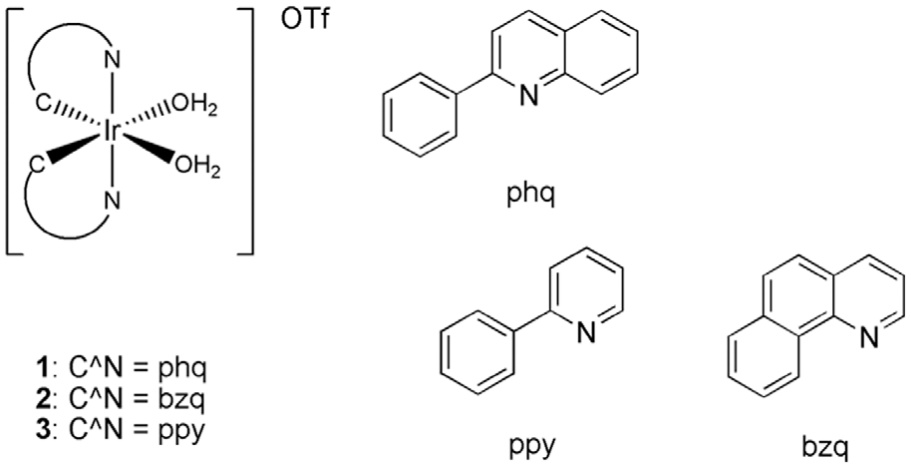
Chemical structures of iridium(III) solvato complexes 1–3 bearing different C^∧^N ligands.

**Figure 2:**
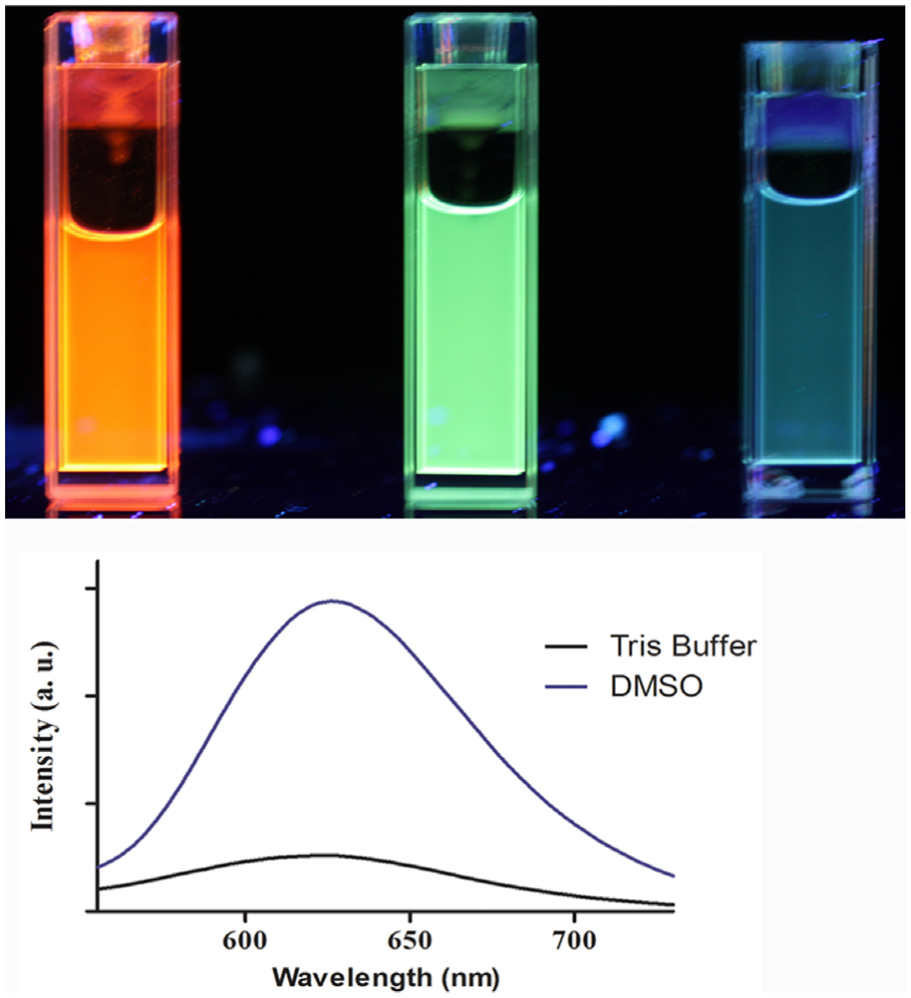
Luminescence photographs (upper panel) of (left) [Ir(phq)_2_(H_2_O)_2_)]OTf (1), (middle) [Ir(ppy)_2_(H_2_O)_2_]OTf (3), and (right) [Ir(bzq)_2_(H_2_O)_2_]OTf (2) at 1 mM concentration in DMSO solution under UV-transillumination. Emission spectra (lower panel) of complex 1 (50 µM) in 20 mM Tris buffer (pH 7.4) and DMSO.

The cyclometalated iridium(III) solvent complexes 1–3 (Figure 1) were synthesized according to previously reported methods (see Materials and Methods). To examine the effect of varying the extent of the conjugation of the C^∧^N co-ligand on the emissive color of the complexes, we first obtained luminescence photographs of the complexes in dimethyl sulfoxide (DMSO) (Figure 2A). Interestingly, complex 1 emits an intense orange luminescence in DMSO under UV-transillumination and was thus considered as a promising candidate for further cell imaging studies. On the other hand, luminescence of 1 was significantly suppressed in Tris buffer (Figure 2B). We rationalize that the reduced luminescence intensity of 1 in aqueous solution is due to non-radiative decay of the excited state of complex 1 by complex-solvent interactions. Presumably, this effect is less pronounced in DMSO, leading to a higher luminescence signal.

**Figure 3:**
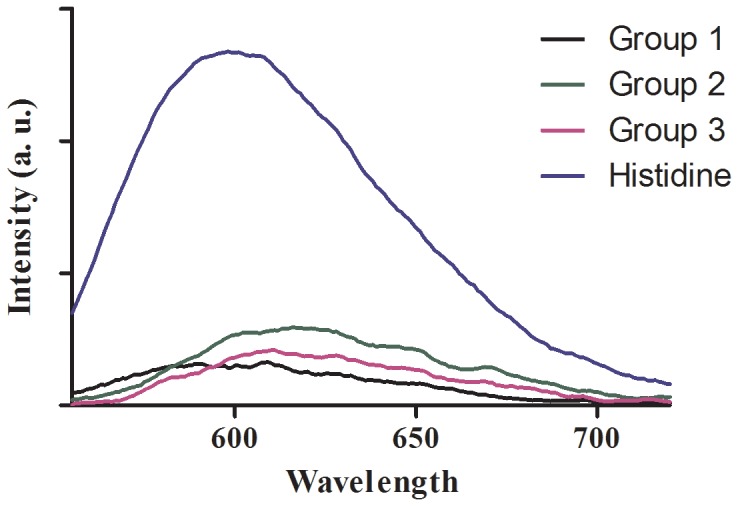
Emission spectra of complex 1 (50 µM) in 20 mM Tris buffer (pH 7.4) with various natural amino acids (200 µM). Group 1: L-alanine, L-arginine, L-asparagine, L-glutamine, L-threonine; Group 2: L-glycine, L-isoleucine, L-lysine, L-phenylalanine, L-proline, L-serine; Group 3: L-tryptophan, L-tyrosine, L-valine, L-glutamic acid, L-cysteine, L-methionine.

**Figure 4:**
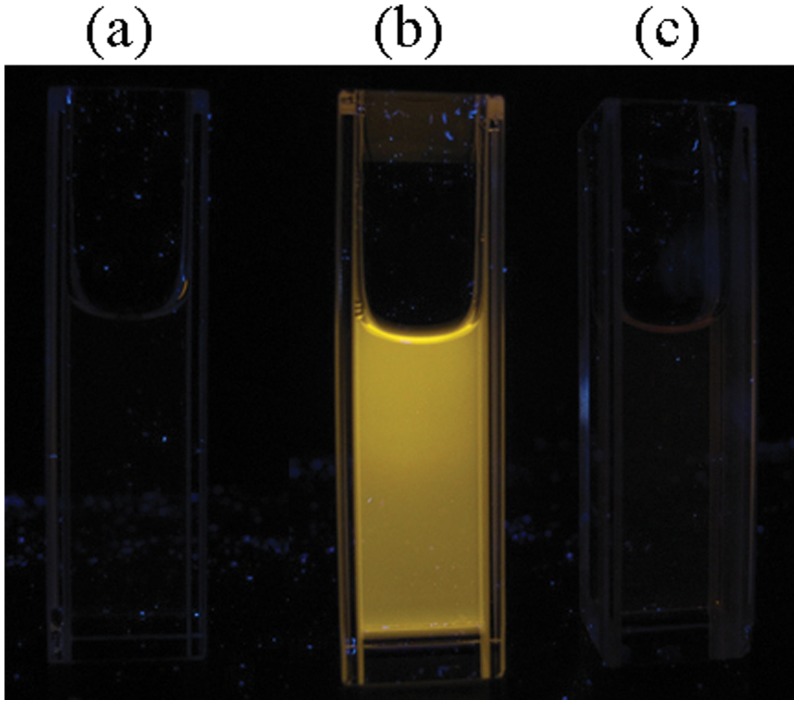
Photograph image of complex 1 (50 µM) in absence (left) or presence of BSA (50 µM, middle) or ct DNA (50 µM, right) under UV-transillumination.

**Figure 5:**
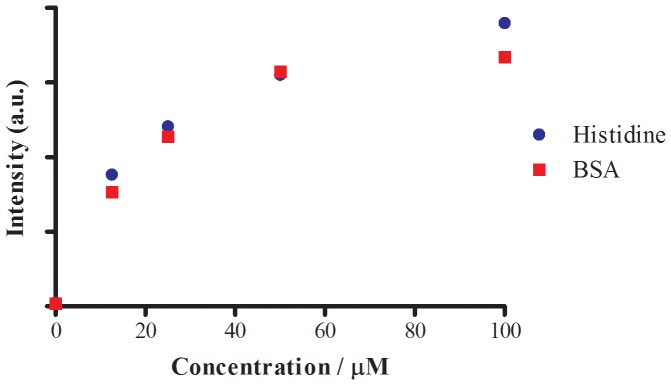
Luminescence intensity changes of complex 1 (50 µM) in 20 mM Tris buffer (pH 7.4) with various amounts of BSA or histidine (0, 12.5, 25, 50 and 100 µM).

We also investigated the application of iridium(III) complex 1 for staining fixed cells. HeLa cells fixed with 4% paraformaldehyde exhibited strong intracellular luminescence in the cytoplasm upon incubation with complex 1 (Figure 8b). Similar to the results with live cells, only weak luminescence was observed in the nucleus of the fixed cells (Figure 8c,d). These results suggest that complex 1 is an effective luminescent cytoplasmic stain for both living and dead cells.

**Figure 6:**
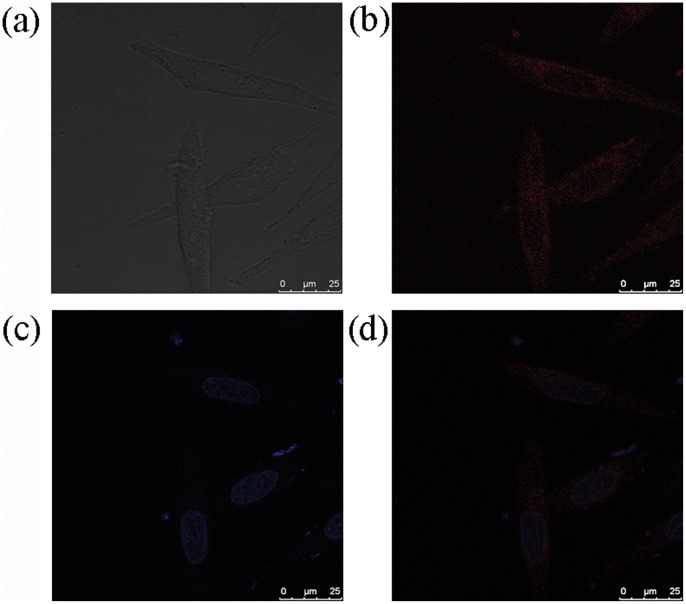
Brightfield images of live HeLa cells (top left). Luminescence images of cells stained with complex **1** (10 µM) in DMSO/PBS (pH 7.4, 1∶99 v/v) for 10 min at 37°C (top right) and then with Hoechst 33258 for a further 20 min (bottom left). Overlay of images in (b) and (c) (bottom right).

**Figure 7:**
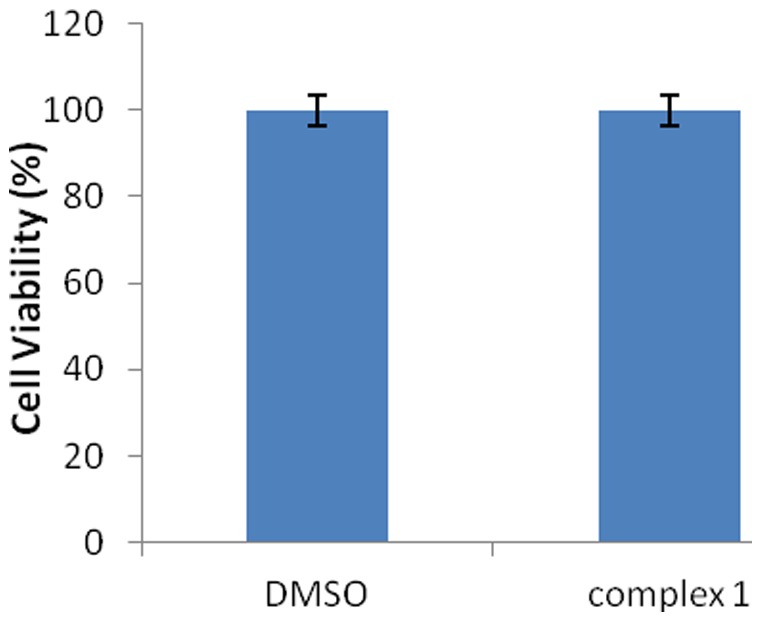
Cytotoxicity of complex 1 (concentration of 1 = 10 µM; incubation time  = 10 min).

The practical application of complex 1 as a luminescent probe in living cells was investigated using confocal laser scanning microscopy (Figure 6). HeLa cells showed negligible background fluorescence. After incubation with 10 µM of 1 in DMSO/PBS (pH 7.4, 1∶99, v/v) for 10 min at 37°C, an intense intracellular luminescence was observed particularly in the cytoplasm of the cells, suggesting that the iridium(III) complex is cytoplasmic permeable. No cell death was observed under the staining and imaging conditions used (Figure 7). Overlay images revealed that the luminescence pattern of complex 1 differed considerably from that of DNA-binding dye Hoechst 33258 (Figure 6d). Furthermore, a large signal ratio was observed between the nuclei and cytoplasm, indicating that complex 1 prefers to stain the cytoplasmic regions of the cells. We presume that the observed luminescence enhancement of complex 1 is due to its interactions with histidine or histidine-rich proteins in the cellular cytoplasm. These results indicate that complex 1 acts as a luminescent imaging agent for live cells without requiring prior membrane permeabilization.

**Figure 8:**
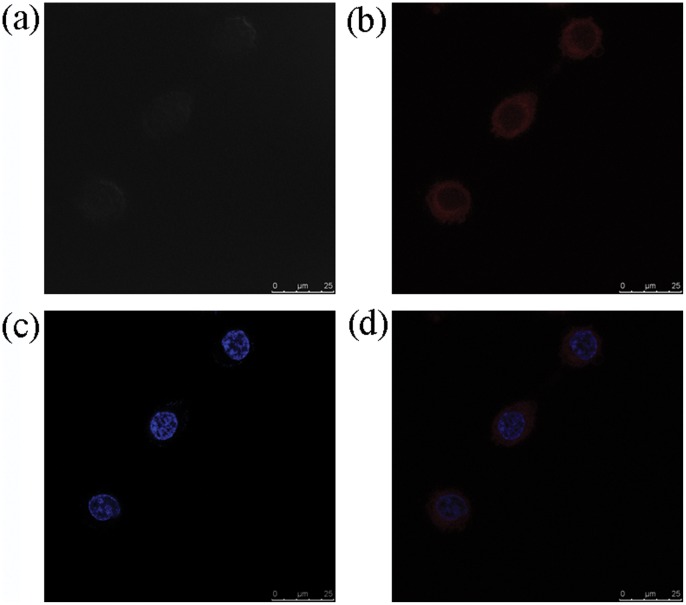
Brightfield images of fixed HeLa cells (top left). Luminescence images of cells stained with complex **1** (10 µM) in DMSO/PBS (pH 7.4, 1∶99 v/v) for 10 min at 37°C (top right) and then with Hoechst 33258 for a further 20 min (bottom left). Overlay of images in (b) and (c) (bottom right).

### Conclusions

In conclusion, we have presented the cytoplasmic permeable iridium(III) complex 1 as a phosphorescent dye for live and fixed cell imaging. Complex 1 shows a bright phosphorescence in living cells, and effectively enters and stains the cytoplasm. Given that the emission properties of metal complexes can be fine-tuned through modifications of auxiliary ligands, we envision that further improvements can be achieved in the application of luminescent iridium(III) complexes as cellular imaging probes.

## Materials and Methods

### Materials

Iridium chloride hydrate (IrCl_3_.xH_2_O) was purchased from Precious Metals Online. DMSO, L-alanine, L-arginine, L-asparagine, L-glutamine, L-glycine, L-isoleucine, L-lysine, L-phenylalanine, L-proline, L-serine, L-threonine, L-tryptophan, L-tyrosine, L-valine, L-glutamic acid, L-cysteine, L-methionine, L-histidine, bovine serum albumin, and calf thymus DNA were obtained from Sigma (St. Louis, MO). Hoechst 33258 and cell culture reagents were purchased from Invitrogen (Carlsbad, CA).

### Synthesis of [Ir(C^∧^N)_2_(H_2_O)_2_]OTf (1–3, where OTf = trifluoromethanesulfonate)

The following complexes were prepared using literature methods: [Ir_2_(ppy)_4_Cl_2_] [77], [Ir_2_(bzq)_4_Cl_2_] [77], [Ir_2_(phq)_4_Cl_2_] [78], [Ir(ppy)_2_(H_2_O)_2_]OTf [79], [Ir(phq)_2_(H_2_O)_2_]OTf [76], and [Ir(bzq)_2_(H_2_O)_2_]OTf [76].

### Emission Measurement

A stock solution of the complex [Ir(phq)_2_(H_2_O)_2_)]OTf was diluted (50 µM, final concentration) into Tris buffer (20 mM, pH 7.4) with the corresponding concentrations of histidine (200 µM), groups of other amino acids (200 µM), ct DNA (50 µM) or BSA (50 µM). The emission spectra were recorded in the 555–730 nm range, after equilibration at 25°C for 5 min. Excitation wavelength = 365 nm.

### Cell Culture

HeLa cells were maintained in minimum essential medium (MEM) supplemented with fetal bovine serum (10%), penicillin (100 U mL^−1^), streptomycin (100 µg mL^−1^) at 37°C under a humidified atmosphere with 5% CO_2_.

### Luminescence Imaging

For colocalization imaging of living cells. The cells were washed with PBS, then incubated with 10 µM of iridium complex in DMSO/PBS (pH 7.4, 1∶99, v/v) for 10 min at 37°C, and then further incubated with Hoechst 33258 for another 20 min before imaging.

For colocalization imaging of fixed cells. The cells were detached from the culture and were fixed with 4% paraformaldehyde at room temperature for 20 min. After washing with PBS, the fixed cells were incubated with 10 µM of iridium complex in DMSO/PBS (pH 7.4, 1∶99, v/v) for 10 min at 37°C, and then further stained with Hoechst 33258 for another 20 min. After washing with PBS, the coverslips were separated from the chamber, and the cells were mounted with 10% glycerol and sealed with nail varnish on a glass substrate.
